# Complete Genome Sequences of Genamy16 and NovaSharks, Two Gordonia rubripertincta Bacteriophages Isolated from Soil in Southeastern Florida

**DOI:** 10.1128/mra.00973-22

**Published:** 2022-11-03

**Authors:** Julie Torruellas Garcia, Sarah Ballarin, Neel Balusa, Melissa Bell, Samia Caballero, Joshua Chan, Maria Farez, Ashley Guillen-Tapia, Kristin Parent, Nashrah Pierre-Louis, Victoria Polishuk, Bhavya Soni, Sundharraman Subramanian, Katie Crump

**Affiliations:** a Department of Biological Sciences, Nova Southeastern University, Fort Lauderdale, Florida, USA; b Department of Psychology and Neuroscience, Nova Southeastern University, Fort Lauderdale, Florida, USA; c H. Wayne Huizenga College of Business and Entrepreneurship, Nova Southeastern University, Fort Lauderdale, Florida, USA; d Department of Biochemistry and Molecular Biology, Michigan State University, East Lansing, Michigan, USA; Queens College CUNY

## Abstract

We report on two actinobacteriophages, Genamy16 and NovaSharks, that were isolated from soil in Florida using Gordonia rubripertincta NRRL B-16540. The genomes of both phages are ~65,000 bp, with similar GC contents, and, based on gene content similarity to phages in the Actinobacteriophage Database, were assigned to phage cluster DV.

## ANNOUNCEMENT

Bacteriophages (phages) have been used as a tool in many sectors to prevent bacterial growth. In the food industry, phages are applied to food as nonchemical means to prevent food spoilage by bacteria (1). With the rise of antimicrobial resistance, phage therapy is a promising alternative to conventional antibiotics (2). Additionally, phage treatment is being explored as a method for bioremediation of oil spills and wastewater treatment (3). Here, we report the discovery of two new phages, Genamy16 and NovaSharks, that infect Gordonia rubripertincta, a Gram-positive soil bacterium that can break down hydrocarbons (4).

**FIG 1:**
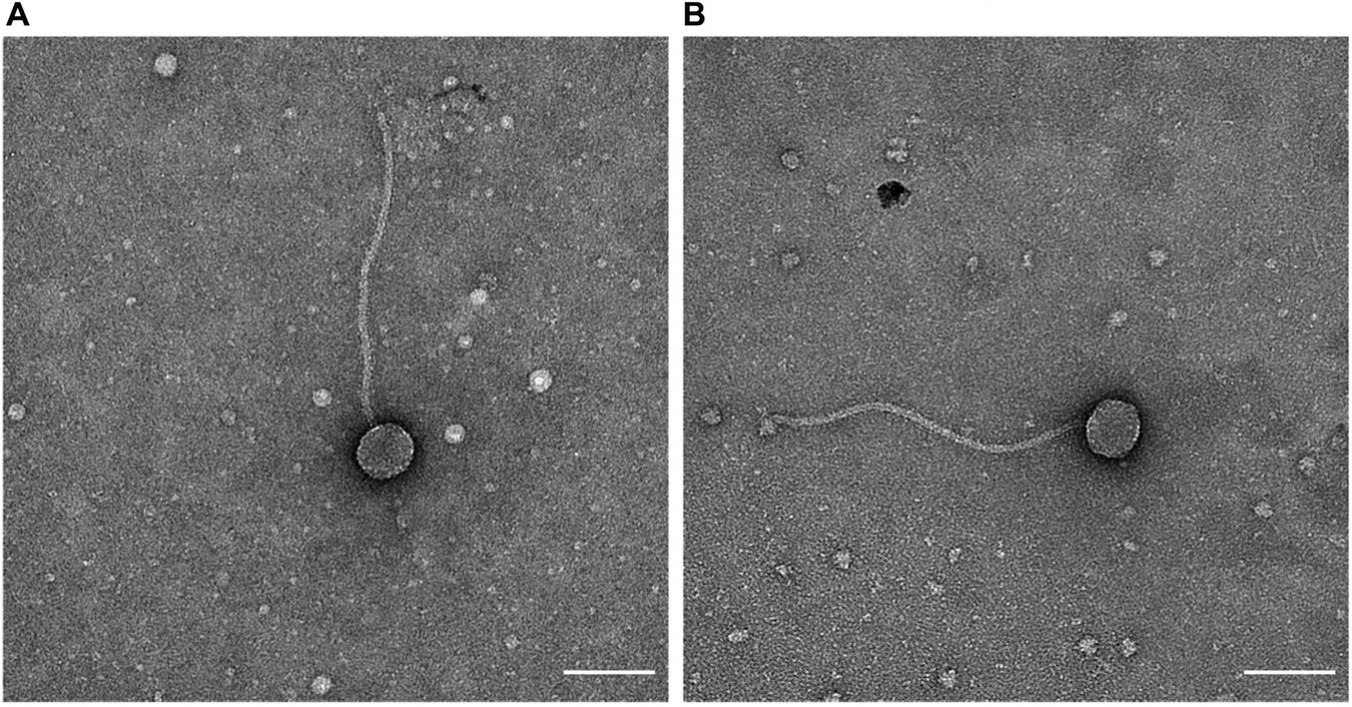
Virion morphologies of Gordonia rubripertincta phages Genamy16 and NovaSharks. High-titer phage lysates of Genamy16 (A) and NovaSharks (B) were prepared for transmission electron microscopy. Continuous carbon support film grids were glow discharged (PELCO easiGlow, 15 mA) for 45 s, and the samples were applied to the grids and incubated for 60 s. The grids were then washed with distilled water and stained with 1% aqueous uranyl acetate. The samples were imaged at the RTSF Cryo-EM Core Facility at Michigan State University using a Talos Arctica system operated at 200 keV. Micrographs were collected with a Ceta camera at a nominal magnification of ×57,000 (1.78 Å/pixel), with an exposure time of 1.0 s and a lens objective defocus setting of 5-μm underfocus. Scale bar = 100 nm.

Both phages were isolated using standard methods (5). Soil samples that had been collected at Nova Southeastern University (Davie, FL, USA) (Table 1) were washed in peptone-yeast-calcium (PYCa) medium, and the wash was then filtered (0.22-μm pore size). A fraction of the filtrate was plated in top agar with Gordonia rubripertincta NRRL B-16540 and incubated at 30°C, yielding phage Genamy16. The remaining filtrate was inoculated with Gordonia rubripertincta and incubated with shaking at 30°C for 5 days before being filtered and plated in top agar with Gordonia rubripertincta, yielding phage NovaSharks. Both bacteriophages were purified via three rounds of plating and exhibited small, clear plaques (approximately 1.0 to 1.5 mm in diameter) after incubation at 30°C for 72 to 96 h. Negative-staining transmission electron microscopy demonstrated a *Siphoviridae* morphotype for both phages (Fig. 1A and B). The tail length and capsid diameter for each phage are shown in Table 1.

**TABLE 1:** Sample isolation location, phage size, and genomic characteristics for Genamy16 and NovaSharks

Phage name	Soil collection site coordinates	Capsid diam (nm)	Tail length (nm)	Sequencing read type	No. of reads	Avg sequencing coverage (×)	Cluster	Genome size (bp)	Genome ends	GC content (%)	No. of genes
Genamy16	26.081101N, 80.243822W	63.8 ± 0.92 (*n* = 5)	355.7 ± 12.3 (*n* = 5)	150-base single end	580,092	1,257	DV	65,574	Circularly permuted	57.6	97
NovaSharks	26.076111N, 80.243306W	65.2 ± 1.44 (*n* = 5)	363.9 ± 5.39 (*n* = 5)	150-base single end	464,243	1,017	DV	65,274	Circularly permuted	57.7	96

Genomic DNA was isolated using the Wizard DNA Clean-Up System (Promega). The Pittsburgh Bacteriophage Institute prepared sequencing libraries with the NEBNext Ultra II Library Kit (New England Biolabs). The libraries were run on an Illumina MiSeq instrument (v3 reagents). For each phage, the number of reads, read length, and coverage are shown in Table 1. Reads were assembled using Newbler v2.9 and quality checked for coverage and genome termini using Consed v29.0 as described previously (6, 7). Table 1 details the genomic characteristics for both phages. Genamy16 and NovaSharks were assigned to phage cluster DV based on content similarity of at least 35% to phages within the Actinobacteriophage Database (8, 9).

Both genome sequences were autoannotated using DNA Master v5.23.6 (10) embedded with GeneMark v2.5 (11) and Glimmer v3.02 (12) and then were manually refined using Starterator v474 (8). Gene function was assessed with HHpred (13, 14), NCBI BLASTp (15), and SOSUI (16). tRNA genes were assessed with tRNAscan-SE v2.0 (17) and ARAGORN v1.2.38 (18). All software was used with default parameters. No tRNAs were identified in either phage.

The numbers of predicted genes for both phages are detailed in Table 1. All genes are transcribed rightward. The two phages exhibit similar gene organization, with the left arm of the genome containing structure and assembly genes such as the portal protein, minor and major capsid proteins, head-to-tail stopper, tail terminator, major tail protein, and tape measure protein, followed by several minor tail proteins. The right arm of the genome encodes proteins for DNA metabolism functions, including DNA helicase, RecA-like DNA recombinase, oxidoreductase, DnaE-like polymerase III, MazG-like protein, thymidylate synthase, and resolvase.

### Data availability.

The sequence of Genamy16 is available in GenBank with accession no. ON755185 and Sequence Read Archive (SRA) accession no. SRX14443507. The sequence of NovaSharks is available in GenBank with accession no. ON755187 and SRA accession no. SRX14483224.
